# DNA methylation regulates TMEM16A/ANO1 expression through multiple CpG islands in head and neck squamous cell carcinoma

**DOI:** 10.1038/s41598-017-15634-9

**Published:** 2017-11-09

**Authors:** Andrey Finegersh, Scott Kulich, Theresa Guo, Alexander V. Favorov, Elana J. Fertig, Ludmila V. Danilova, Daria A. Gaykalova, Joseph A. Califano, Umamaheswar Duvvuri

**Affiliations:** 1 Department of Otolaryngology, University of Pittsburgh School of Medicine, Pittsburgh, PA USA; 2 Division of Otolaryngology-Head and Neck Surgery, Department of Surgery, University of California, San Diego, CA USA; 3 VA Pittsburgh Healthcare System, Pittsburgh, PA USA; 4 Department of Otolaryngology-Head and Neck Surgery, Johns Hopkins University School of Medicine, Baltimore, MD USA; 5 Department of Oncology, Johns Hopkins University School of Medicine, Baltimore, MD USA; 6 Laboratory of Systems Biology and Computational Genetics, Vavilov Institute of General Genetics, RAS, Moscow, Russia

## Abstract

ANO1 is a calcium-activated chloride channel that is frequently overexpressed in head and neck squamous cell carcinoma (HNSCC) and other cancers. While ANO1 expression negatively correlates with survival in several cancers, its epigenetic regulation is poorly understood. We analyzed HNSCC samples from TCGA and a separate dataset of HPV+ oropharyngeal squamous cell carcinoma (OPSCC) samples to identify differentially methylated regions. E6 and E7 transfected normal oral keratinocytes (NOK) were used to induce hypermethylation of the ANO1 promoter. We found three CpG islands that correlated with ANO1 expression, including two positively correlated with expression. Using two HNSCC datasets with differential expression of ANO1, we showed hypermethylation of positively correlated CpG islands potentiates ANO1 expression. E7 but not E6 transfection of NOK cells led to hypermethylation of a positively correlated CpG island without a change in ANO1 expression. ANO1 promoter methylation was also correlated with patient survival. Our results are the first to show the contribution of positively correlated CpG’s for regulating gene expression in HNSCC. Hypermethylation of the ANO1 promoter was strongly correlated with but not sufficient to increase ANO1 expression, suggesting methylation of positively correlated CpG’s likely serves as an adjunct to other mechanisms of ANO1 activation.

## Introduction

Head and neck squamous cell carcinoma (HNSCC) is clinically heterogeneous and prevalent, with an estimated 600,000 new cases per year worldwide^1^. Risk factors for HNSCC include environmental factors like ethanol and smoking as well as the oncogenic human papilloma virus (HPV)^2^. Recent advances in treatment of HNSCC and understanding of molecular mechanisms^3–5^ have led to modest improvements in 5-year survival, which remains near 50%^6,7^.

Anoactin-1 (ANO1, TMEM16A, DOG1) was initially identified as a component of the chr11q13 chromosomal band that is frequently amplified in HNSCC, bladder, and breast cancer^8–10^ and has recently been characterized as a calcium-activated chloride channel (CaCC)^11^. ANO1 was shown to activate the Ras-Ref-MEK-ERK pathway to promote tumor proliferation *in vitro* and *in vivo* ^12^. Additionally, differential expression of ANO1 was found to regulate epithelial to mesenchymal transition (EMT) via interactions with Radixin^13^ and depends on DNA methylation at a CpG island near its transcriptional start site (TSS)^13,14^. Overexpression of ANO1 was also found to have preferential effects on tumorigenesis in HPV− cell lines, suggesting inhibiting ANO1 in this population could represent a new treatment avenue^14^.

While DNA methylation has been shown to play a role in regulation of ANO1, specific mechanisms and regions are not yet defined. Notably, the UCSC genome browser annotates three CpG islands near the ANO1 transcriptional start site (TSS)^15^, suggesting multiple possible epigenetic mechanisms regulating expression. Additionally, alternative splicing of ANO1 exons alters CaCC function^16–18^, indicating importance of intragenic sequences. Traditional studies of DNA methylation have focused on CpG islands at promoters of the majority of genes, which are hypermethylated to repress gene expression^19^. However, the majority of CpG’s occur outside of these promoter CpG islands and have inverse effects on expression, with hypermethylation generally promoting expression^20^. These gene body CpG’s were recently shown to regulate expression of a distinct subset of genes in colorectal and prostate cancer^21,22^.

The goal of this study was to identify how DNA methylation regulates expression of ANO1. We utilized two independent epigenomic datasets with differential expression of ANO1 to establish the contribution of individual CpG’s on ANO1 expression. Results from The Cancer Genome Atlas (TCGA) and an additional independent dataset of oropharyngeal squamous cell carcinoma (OPSCC) tumors reveal that, contrary to reports by prior studies, ANO1 expression is primary regulated by positively correlated CpG’s. We also show that transfection of cells with the HPV protein E7 leads to hypermethylation of positively correlated CpG’s without a detectable change in ANO1 expression. These results are expected to have important contributions for studying regulation of ANO1 in both carcinogenesis and cancer treatment.

## Results

### DNA methylation is correlated with ANO1 expression at three CpG islands

**Figure 1:**
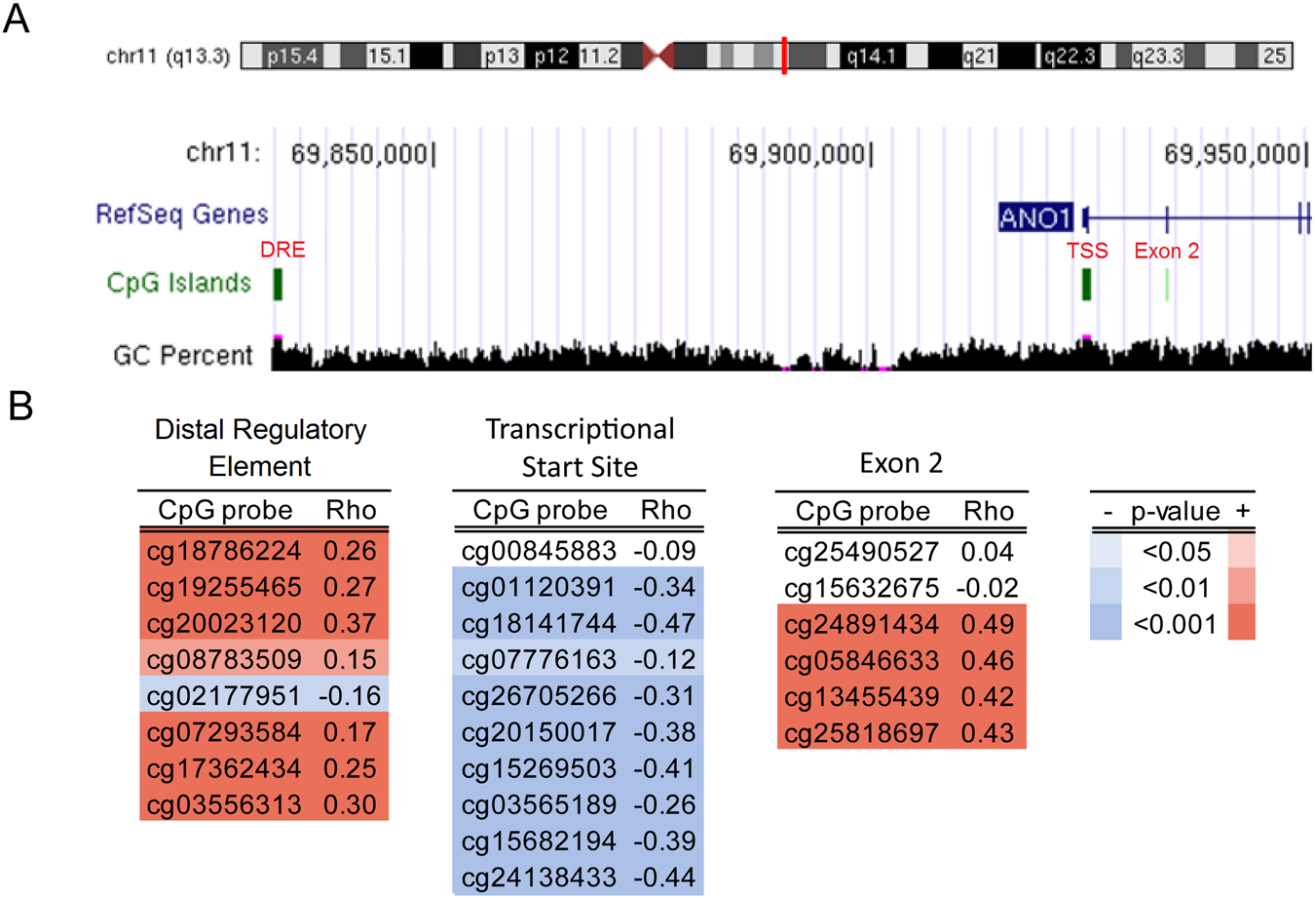
DNA methylation is positively and negatively correlated with expression at the ANO1 promoter. (**A**) We studied three CpG islands annotated by the UCSC Genome Browser indicated in red as the distal regulatory element (DRE), the transcriptional start site (TSS), and exon 2. (**B**) Spearman ρ correlation coefficients for DNA methylation probes versus ANO1 expression show a cluster of positively correlated CpG’s in the DRE and exon 2 and a cluster of negatively correlated CpG’s at the TSS.

The UCSC Genome Browser identified three CpG islands near the ANO1 transcriptional start site (TSS) (Fig. 1A). Using HNSCC tumor samples from TCGA, we correlated DNA methylation at individual CpG’s within these regions with ANO1 expression (Fig. 1B). A CpG island ~90 kb upstream from the TSS previously characterized as a 5′ distal regulatory element (DRE) for a new ANO1 exon^18^ contained CpG’s that were significantly positively correlated with gene expression. A CpG island at the TSS was significantly negatively correlated with expression. Finally, an intragenic CpG island near exon 2 was also found to be significantly positively correlated with gene expression.

### ANO1 expression is regulated by differential methylation of positively correlated CpG islands

**Figure 2:**
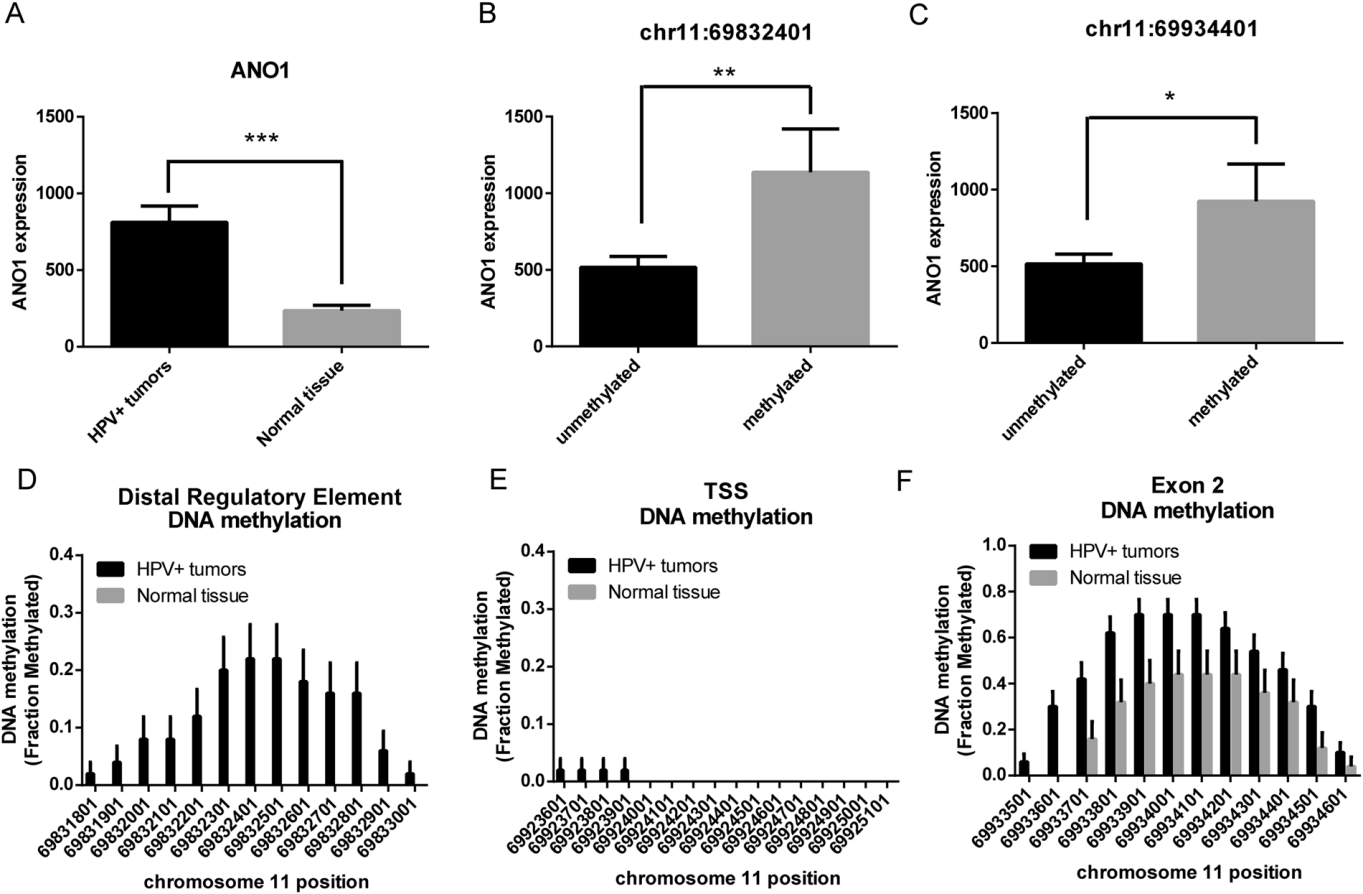
HPV+ OPSCC has increased ANO1 expression relative to normal tissue and increased DNA methylation at positively correlated CpG islands. We studied HPV+ OPSCC compared to normal tissue. (**A**) HPV+ OPSCC has increased expression of ANO1 relative to normal tissue. Samples with DNA methylation at (**B**) the DRE or (**C**) exon 2 CpG island had increased expression of ANO1 compared to those that were unmethylated. HPV+ samples had (**D**) significantly increased DNA methylation at the DRE, (**E**) no difference at the CpG island, and (**F**) significantly increased DNA methylation at the exon 2 CpG island. Data presented as mean ± SEM. *p < 0.05, **p < 0.01, ***p < 0.001.

Given bidirectional correlation of DNA methylation with ANO1 expression across a large segment of the ANO1 promoter, we identified the specific contribution of individual CpGs using two datasets with differential expression of ANO1. We first utilized an independent dataset of HPV+ oropharyngeal squamous cell carcinoma (OPSCC)^23^ in which tumors had significantly greater expression of ANO1 relative to normal oral mucosa (Fig. 2A). Samples with methylation at the DRE and exon 2 CpG islands had significantly greater expression of ANO1 relative to those without methylation (Fig. 2B,C, Supplementary Fig. 1). Additionally, OPSCC samples had significant hypermethylation at the DRE and exon 2 CpG islands with no difference in methylation at the TSS CpG island (Fig. 2D–F). Notably, only one of seventy-five analyzed samples had methylation at the TSS CpG island, indicating this is completely hypomethylated at baseline in both HPV+ OPSCC and normal oral mucosa. DNA methylation at ANO1 was analyzed for differentially methylated regions that drive ANO1 expression. The DRE (chr11:69831572–69832484) and exon 2 (chr11:69933920–69934209) were identified as differentially methylated but differential methylation was not detected at the canonical CpG island at the TSS (chr11:69924401–69925001).

ANO1 expression is driven by gene amplification of chr11q13 in HNSCC^9^, which could also account for increased expression in the OPSCC dataset. Notably, cyclin D1 (CCND1) and cortactin (CTTN), neighboring genes on the chr11q13, were significantly downregulated and unchanged, respectively in HPV+ OPSCC tumors compared to normal tissue (Supplementary Fig. 2), indicating increased ANO1 expression in this dataset was unlikely to be driven by gene amplification of chr11q13.

The CpG island studied at the 5′ DRE was reported to be a promoter for a new ANO1 exon upstream of the exon 1 TSS^17,18^. Therefore, DNA methylation at the 5′ DRE may be acting to regulate transcription initiation at the new exon. Using RNA-seq of OPSCC and normal oral mucosa, we found that only 2 of 72 samples had IGV read counts > 5 for the new exon and only ten tumors had detectable expression (IGV read counts > 1) (Supplementary Fig. 3). Of the 10 tumors with detectable expression, there was no significant difference in DNA methylation at the 5′ DRE after FDR correction. This finding suggests DNA methylation at this region is not associated with expression of this exon.

### Differential methylation of HNSCC tumors and control tissue at the ANO1 promoter

**Figure 3:**
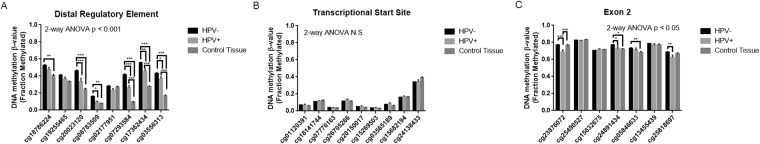
Increased ANO1 expression in HPV− samples is associated with hypermethylation at positively correlated CpG’s relative to HPV+ and control tissue samples. DNA methylation in HPV+ (n = 34) and HPV− (n = 241) tumor samples as well as paired control tissue (n = 48) was compared. Using a two-way repeated measures ANOVA we found (**A**) a significant group effect at the DRE with Bonferroni post-hoc tests revealing hypermethylation at multiple CpG’s in HPV− tumors relative to HPV+ tumors and control tissue as well as HPV+ tumors relative to control tissue, (**B**) no group effect at the TSS, and (**C**) a significant group effect at exon 2 with Bonferroni post-hoc tests revealing hypermethylation at multiple CpG’s in HPV− tumors relative to HPV+ tumors and control tissue. Data presented as mean ± SEM. *p < 0.05, **p < 0.01, ***p < 0.001.

Since the OPSCC dataset was restricted to tumors within the oropharynx with detectable HPV transfection, we validated the relationship between positively correlated CpGs and ANO1 expression using a more representative subset of HNSCC within TCGA^5^. Notably, a recent study using this TCGA dataset showed that HPV− tumors (n = 241) had significantly increased ANO1 expression compared to HPV+ tumors (n = 34) and control head and neck tissue (n = 48)^14^. We utilized differential expression of ANO1 to study which CpGs contribute to increased ANO1 expression in HPV− tumors. There was a significant group effect on DNA methylation at the DRE (p < 0.001) and exon 2 (p < 0.05) CpG islands but not at the TSS CpG island. Bonferroni post-hoc tests were used to study main effects between groups and at individual CpGs. At the DRE CpG island, there was a significant main effect for hypermethylation of HPV− compared to HPV+ tumors (p < 0.001) and control tissue (p < 0.001) as well as a significant main effect for hypermethylation of HPV+ tumors compared to control tissues (p < 0.001). At the exon 2 CpG island, there was a nonsignificant trend for a main effect for hypermethylation of HPV− compared to HPV+ tumors (p = 0.06). There were no significant main effects at the TSS CpG island (Fig. 3A–C).

Within the TCGA dataset, HPV− tumors have overexpression of neighboring chr11q13 genes CTTN and CCND1 relative to HPV+ tumors, suggesting that differential methylation of positively correlated CpG islands may be a feature of other genes on the amplified chromosomal band chr11q13. We compared DNA methylation of HPV+ and HPV− tumors at positively and negatively correlated CpGs within the CTTN and CCND1 promoters. HPV− samples had hypomethylation of negatively correlated CpGs with no change in methylation of positively correlated CpG’s compared to HPV+ samples (Supplementary Fig. 4). This suggests that differential methylation of positively correlated CpG islands is a unique feature of the ANO1 promoter.

**Figure 4:**
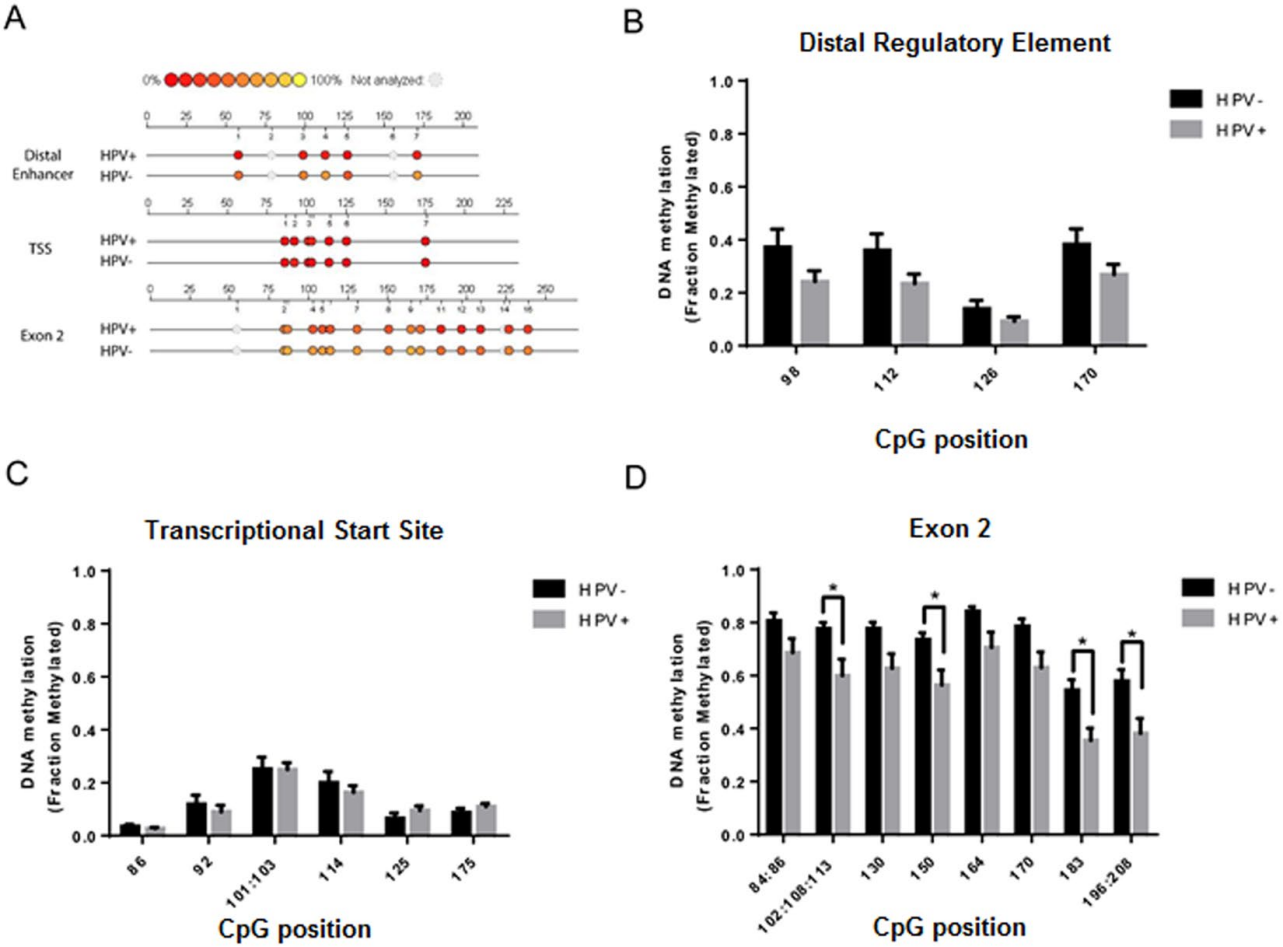
EpiTYPER of HPV+ and HPV− tumor samples validates hypermethylation of positively correlated CpG islands in HPV− tumors. MALDI-TOF mass spectrometry was used to study DNA methylation in HPV− (n = 12) and HPV+ (n = 11) tumor samples. (**A**) Representative Epigram from individual HPV− and HPV+ tumors indicating position of CpG’s within PCR transcript. HPV− tumors had (**B**) a trend for hypermethylation of the DRE, (**C**) no change at the canonical CpG island, and (**D**) significant hypermethylation of the exon 2 CpG island. Data presented as mean ± SEM. *p < 0.05.

Differences in DNA methylation at the ANO1 promoter in HPV− and HPV+ TCGA samples were validated with additional tumor samples using EpiTYPER, a quantitative DNA methylation tool utilizing MALDI-TOF mass spectrometry. DNA methylation was characterized at multiple CpG’s within each CpG island (Fig. 4A). HPV− samples (n = 12) had a nonsignificant trend toward hypermethylation of the DRE (p = 0.09), no difference in DNA methylation at the TSS, and significant hypermethylation of the exon 2 CpG island (p < 0.05) compared to HPV+ samples (n = 11) (Fig. 4B–D). These findings parallel those using the Illumina 450 K DNA methylation in TCGA.

### Copy number variation and DNA methylation

Gene amplification of chr11q13 is a feature of HPV− HNSCC^24^; therefore, we analyzed the effect of copy number variation of ANO1 on DNA methylation in the TCGA dataset. Paradoxically, tumor samples with ANO1 amplification had potentiating effects of DNA methylation, with significant hypermethylation at positively correlated CpG’s and significant hypomethylation at a negatively correlated CpG (Supplementary Fig. 5).

### E6 and E7 transfection on ANO1 DNA methylation and expression

**Figure 5:**
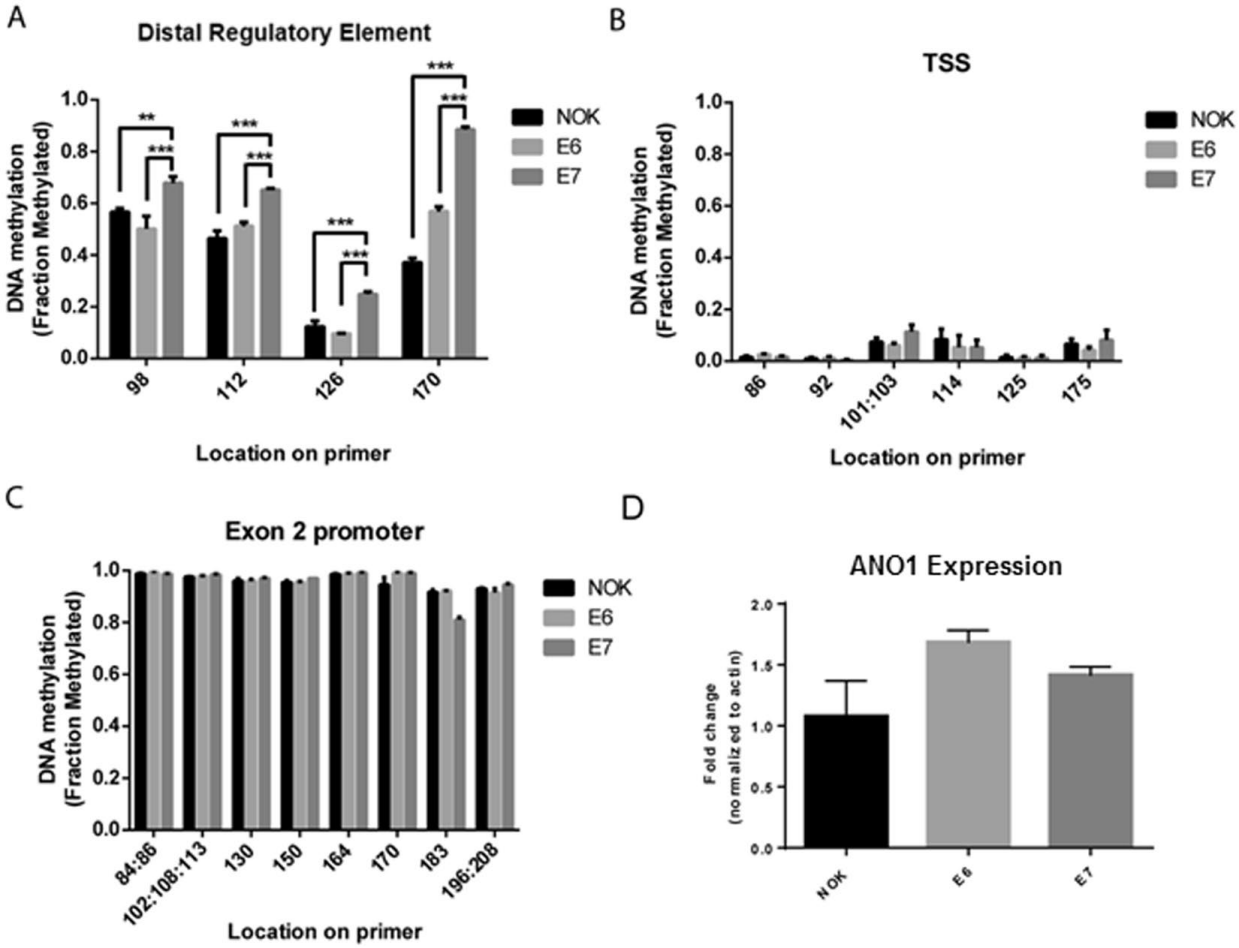
E7 transfection hypermethylates the ANO1 DRE CpG island but does not affect gene expression. EpiTYPER was used to analyze DNA methylation in NOK cell lines transfected with E6 (n = 3), E7 (n = 3), or empty vector control (n = 3) and found (**A**) increased DNA methylation at the DRE in E7-transfected cells but no change in methylation at the (**B**) TSS or (**C**) exon 2. (**D**) There were no significant changes in ANO1 expression after E6 or E7 transfection. Data presented as mean ± SEM. **p < 0.01, ***p < 0.001.

Based on increased DNA methylation in OPSCC HPV+ tumors relative to normal mucosa, we hypothesized that transfection of NOK cells with HPV viral proteins E6 and E7 could induce ANO1 promoter methylation and expression. E7 but not E6 transfection was associated with significant hypermethylation of the DRE CpG island (Fig. 5A). E6 and E7 transfection did not alter in DNA methylation of the TSS and exon 2 or ANO1 expression (Fig. 5B–D), suggesting methylation of the DRE is not sufficient to induce ANO1 expression.

### ANO1 promoter methylation is associated with survival

**Figure 6:**
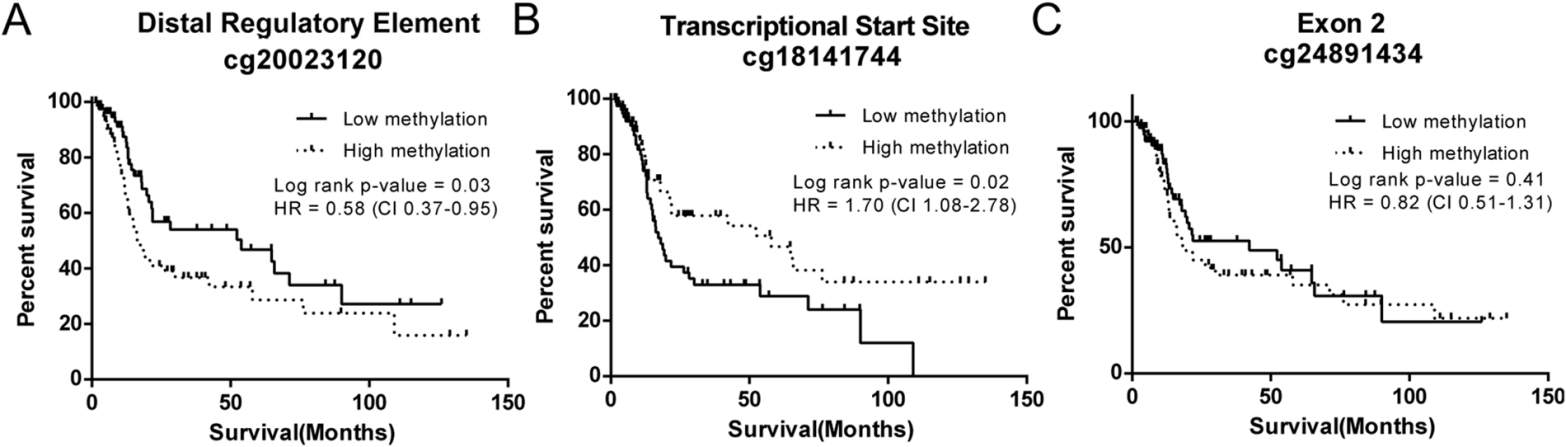
DNA methylation at both positively and negatively correlated CpGs is associated with patient survival. Kaplan-Meier curves were used to assess survival of patients with HNSCC. Patients had significantly worse overall survival with (**A**) increased DNA methylation at the positively correlated CpG cg200232120, (**B**) decreased DNA methylation at the negatively correlated CpG cg18141744, (**C**) but no change in survival at the positively correlated CpG cg24891434. Low versus high was defined as below or above the median, respectively, of all samples studied. HR = Hazard Ratio, CI = 95% Confidence Interval.

TCGA samples with available survival data (n = 160) were used to study the effects of DNA methylation at the ANO1 promoter on patient survival. Illumina probes with the highest Spearman correlation coefficient within each CpG island were chosen for analysis. Subjects were divided into high and low methylation and expression groups using the median β-value for each CpG. Higher expression of ANO1 was previously shown to be associated with decreased survival^12^. Consistent with their correlations with ANO1 expression, hypermethylation of CpG cg200232120 at the DRE is associated with decreased survival (HR 0.58, p < 0.05) (Fig. 6A), hypomethylation of CpG cg18141744 at the TSS is associated with decreased survival (HR 1.70, p < 0.05) (Fig. 6B), (D) but there is no change in survival at CpG cg24891434 at exon 2 (HR 0.82, p = 0.41) (Fig. 6C).

## Discussion

Our results demonstrate a novel mechanism for epigenetic regulation of ANO1. Using TCGA, we identified three CpG islands where DNA methylation was positively or negatively correlated with expression. We found that HPV+ OPSCC tumors had complete hypomethylation of a canonical TSS CpG island that was previously shown to regulate ANO1 expression^13,14^, indicating that the TSS CpG island was likely not a driver for differential expression of ANO1 in these tumors. In this subset of tumors, DNA hypermethylation at positively correlated CpGs was associated with increased ANO1 expression. We validated these findings using a subset of heterogeneous HNSCC tumors from TCGA in which HPV− tumors have significantly greater ANO1 expression and hypermethylation of positively correlated CpGs compared to HPV+ tumors and control tissue. Therefore, two independent datasets were used to show that positively correlated CpGs drive ANO1 expression, revealing this likely as a fundamental mechanism for regulating ANO1 in HNSCC. We also show that E7 transfection of NOK cells induces hypermethylation of positively correlated CpG’s but that a two-fold increase in methylation was not sufficient to increase ANO1 mRNA levels. DNA methylation at the ANO1 promoter in HNSCC was also found to correlate with overall survival, indicating clinical relevance of both negatively and positively correlated CpG’s. To our knowledge, this is the first study to show that DNA hypermethylation is associated with potentiating gene expression in HNSCC.

The role of gene body DNA methylation in cancer is emerging and has been associated with potentiating gene expression in acute myeloid leukemia, prostate carcinoma, and colorectal carcinoma^21,22,25^. Yang *et al*. (2014) showed that 5-aza-2′-deoxycytidine induced DNA demethylation in a colorectal carcinoma cell line down-regulated a subset of genes with positively correlated gene body CpG’s and that re-methylation of these regions was dependent on the DNA methyltransferase DNMT3B^22^. The authors hypothesized that gene body DNA methylation exerted its effects on gene expression through nucleosome destabilization at the intron-exon junction to promote transcription. While our study did not investigate chromatin at the ANO1 promoter, the location of the positively correlated CpG’s at the intron-exon 2 junction of ANO1 supports this idea. Other mechanisms may account for the effect of positively correlated CpG’s ~90 kb upstream of the ANO1 promoter on expression. DNA methylation plays a role in recruitment of polycomb-group proteins and establishment of chromatin architecture to drive expression^26,27^, so that DNA methylation at the ANO1 5′ DRE may recruit these proteins to link it with the ANO1 promoter. Alternatively, DNA methylation at the 5′ DRE may be repressing expression of the recently discovered exon 0^17,18^ to promote transcription initiation at the exon 1 TSS; however, we did not detect significant enrichment in RNA-seq reads near the 5′ DRE in OPSCC, suggesting this region is unlikely to be an exon 0 CpG island. While mechanisms by which gene body DNA methylation promotes expression are still being uncovered, studying chromatin of the ANO1 promoter may offer insights into specific histone modifications and transcription factors involved.

The extent to which gene body DNA methylation affects gene expression is also emerging. Maunakea *et al*. (2010) found that less than 3% of CpG islands near 5′ promoters were methylated in multiple human tissue types, while 34% of intragenic CpG’s were methylated^28^. Similarly, in this study, ANO1’s canonical TSS promoter CpG island was completely unmethylated in OPSCC tumors and normal oral mucosa, with differential methylation of positively correlated CpG’s at the 5′ DRE and exon 2 associated with ANO1 expression. Notably, ~20% of OPSCC tumors acquired *de novo* methylation of the 5′ DRE, indicating hypermethylation of positively correlated CpG’s may be a feature of some HPV+ tumors. While our data shows strong correlations between positively correlated CpG’s and ANO1 expression, establishing a mechanistic is challenging. We found that HPV E7 transfection induces *de novo* DNA methylation of positively correlated CpG’s at the ANO1 5′ DRE; however, this was not associated with a change in ANO1 expression. Interestingly, in the TCGA dataset, HPV+ tumors also had hypermethylation of the 5′ DRE relative to control tissue but no difference in ANO1 expression. Taken together, these findings indicate that methylation of positively correlated CpG’s at the 5′ DRE is not sufficient to drive ANO1 expression. New tools for mechanistic studies of DNA methylation are emerging, including recent improvement of a Crispr-Cas9 driven delivery for TET1 and DNMT3a to specific DNA sequences^29^. Utilizing this system to methylate or demethylate clusters of positively correlated CpG’s will help establish the role of gene body DNA methylation.

Failure of E7-induced hypermethylation of the ANO1 5′ DRE to increase ANO1 mRNA levels suggests modest changes in DNA methylation in this region function as an adjunct to other mechanisms driving ANO1 expression. Interestingly, these results were similar to those seen in the TCGA dataset, where HPV+ tumors had no change in ANO1 expression but increased methylation relative to control tissue. In contrast, HPV− tumors had even greater levels of methylation at the 5′ DRE and substantially increased ANO1 expression. This suggests that there may be a threshold where DNA methylation acts to increase ANO1 expression. Other mechanisms may also be involved. E7 interacts with DNMT1 to stimulate methyltransferase activity^30^, so that E7-induced hypermethylation may prime ANO1 for expression and require other components of the HPV viral genome prior to transcription. Oncogenic pathways specific to HPV− HNSCC may also play a role in potentiating the effects of hypermethylation at the 5′ DRE on ANO1 expression. Using transformed cell lines rather than NOK cells for E6 and E7 transfections may have shown an effect on expression. Additional studies utilizing co-transfection of multiple HPV viral proteins may establish whether HPV transfection plays a causal role on ANO1 expression.

This study builds on a previous study using TCGA to study how DNA methylation affects ANO1 expression. Dixit *et al*. (2015) identified a single CpG within the TSS CpG island that was negatively correlated with expression and found that it was hypomethylated in HPV− compared to HPV+ tumors. The authors validated these findings using quantitative methylation specific PCR (qMSP). Our study expanded on these results by studying all CpG’s near the ANO1 promoter and used multiple comparisons corrections to reduce the chance of type I error. Notably, we did not find a group effect for hypomethylation at the TSS CpG island on ANO1 expression. A prior study by Shiwarski *et al*. (2014) had also indicated treatment with 5-aza increased expression of ANO1 and hypothesized this was due to hypomethylation of the TSS CpG island^13^. While those results seem to contradict those presented in this paper, Shiwarski *et al*. (2014) used a HNSCC cell line for epigenetic experiments, which are unlikely to replicate the *in vivo* tumor environment; additionally, 5-aza likely had downstream effects that may have led to ANO1 potentiation independent of its effects on DNA methylation. Mechanistic effects on DNA methylation may be studied using CRISPR-Cas9 DNMT and TET1 constructs as previously discussed.

Paradoxically, tumors with amplification of ANO1 had potentiation of DNA methylation at multiple CpG’s. This interaction suggests increasing expression of ANO1 and other components of chr11q13 to a level that drives oncogenesis may follow a “two-hit” mechanism requiring copy number amplification and epigenetic disruption^31^. Alternatively, the interaction may represent bias of copy number variation in the DNA methylation assay; however, this is unlikely given hyper- and hypomethylation were both correlated with ANO1 expression. Interactions between DNA methylation and copy number variation have not been well studied. Poage *et al*. (2010) reported global interactions between DNA methylation and copy number in HNSCC but did not demonstrate specific effects at a single gene^32^. Integrative analysis of osteosarcoma tumors also showed a subset of genes that had alterations in gene expression, copy number variation, and DNA methylation, though heterogeneity between tumors limited generalizing across tumors^33^. Unfortunately, whether DNA methylation and copy number amplification are mechanistically linked is unknown and could be studied in the context of ANO1 expression.

Association of ANO1 promoter DNA methylation with patient survival suggests it has utility as a biomarker for prognosis. Promoter methylation biomarkers utilizing serum and saliva have been developed for HNSCC^34,35^, though none are currently used in clinical practice. Notably, E7 transfection led to hypermethylation at the 5′ DRE without a detectable change in expression, indicating that epigenetic changes may precede changes in expression. Therefore, ANO1 promoter methylation may be worth studying biomarker in predicting survival and risk for HNSCC.

In conclusion, we show that ANO1 expression is correlated with CpG’s at three CpG islands annotated by the UCSC genome browser. Based on data from TCGA and a separate database of OPSCC tumors, the contribution of positively correlated CpG’s appears to exert a greater effect on ANO1 expression. We show that transfection of a NOK cell line with the HPV viral protein E7 induces *de novo* methylation of positively correlated CpG’s at the ANO1 5′ DRE, but that this does not increase ANO1 mRNA levels. These results have important implications for understanding regulation of ANO1 expression and how gene body CpG’s exert effects on transcription. Further studies utilizing Crispr-Cas9 mediated TET1 and DNMT3A delivery to positively correlated CpG’s in ANO1 may establish a mechanistic link between DNA methylation and expression.

## Methods

### TCGA analysis

Data was downloaded from a recent epigenomic characterization of 279 HNSCC tumors and control tissue^5^ within TCGA using the TCGA data portal and cBioPortal^36^. DNA methylation was downloaded as β-values representing methylation at a specific probe from the Illumina 450 K DNA methylation array; probe positions were annotated using the UCSC genome browser. Two subjects each from the HPV− tumors, HPV+ tumors, and control tissue were excluded due to incomplete DNA methylation datasets. Gene expression was downloaded as RNA-seq z-scores. Copy number data was downloaded as an RAE algorithm indicating chromosomal amplification, gain, neutrality, or loss^37^. Available patient survival data and HPV status was also downloaded.

Statistical analysis was performed using SPSS (IBM, Endicott, NY) and GraphPad Prism 6 (GraphPad, La Jolla, CA, USA). We calculated a Spearman rank correlation coefficients between DNA methylation and gene expression for all probes annotated to ANO1, CCND1, and CTTN. To assess the effect of HPV infection on DNA methylation, sets of probes were analyzed separately depending on their location within CpG islands (ANO1) or whether they were significantly positively or negatively correlated with expression (CCND1, CTTN). A two-way repeated measures ANOVA with Bonferroni post-hoc tests was used to identify a group effect on DNA methylation. For copy number data, subjects were divided by gain, amplification, neutrality, and loss of ANO1. A one-way ANOVA with Bonferroni post-hoc tests was used to identify an effect of copy number on DNA methylation. Kaplan Meier curves were used to study the effect of ANO1 expression and methylation on patient survival.

### OPSCC analysis

All tissue samples were collected after informed consent from patients under an approved IRB protocol at Johns Hopkins University in accordance to guidelines and regulations of the institution and submitted to the Johns Hopkins Tissue Core. Primary tumor tissue samples were obtained from a test cohort of 50 patients with HPV− related oropharyngeal squamous cell carcinoma (OPSCC) as previously described^23^. For comparison, normal oropharynx mucosal tissue from uvulopalatopharyngoplasty (UPPP) surgical specimens were obtained from 25 controls with no clinical evidence of cancer. Gene expression was calculated from RNA-seq data for 47 HPV+ OPSCC cases and 25 normal controls using RSEM methods^38^ and log transformed. Three tumor samples did not pass RNA quality control and were excluded. Waterfall bar plots were generated by subtracting the median gene expression of normal tissue. Median gene expression in normal and tumor tissue were compared using Wilcoxon test.

Genome-wide DNA methylation analysis was carried out using MBD-Seq similar to what was described previously^39,40^. Briefly, DNA was sonicated, end-repaired, and ligated to SOLiD P1 and P2 sequencing adaptors lacking 5′ phosphate groups, using the NEBNext DNA Library Prep Set for SOLiD according to the manufacturer’s recommended protocol (NEB). Libraries were then nick-translated with Platinum Taq polymerase and divided into two fractions: an enriched methylated fraction that was subjected to isolation and elution of CpG-methylated library fragments by using MBD2-MBD-bound magnetic beads as described previously^40^, and a total input fraction that was left unenriched. These fractions were then amplified using 4–6 cycles for the total input, and 10–12 cycles for the enriched methylated fractions according to the NEBNext DNA Library Prep Set for SOLiD kit (NEB). The resulting libraries were subjected to emulsion PCR, bead enrichment, and sequencing on a SOLiD sequencer to generate on average ~25–50 million 50 bp single-end reads per sample according to the manufacturer’s protocols (Life Tech). The resulting color-space reads were aligned using Bioscope software (Applied Biosystems).

Utilizing MACS-processed MBD-Seq data, we separated the human genome into 30,975,368 of 100 bp regions. The methylation status of each 100 bp segment for each sample was identified by MACS peak calling^41^. For each 100 bp region, we calculated the Fisher Exact test p-values between cases and controls (47 HPV+ OPSCC cases and 25 normal controls). All the regions with Fisher Exact test p-value > 0.05 after FDR correction were considered significantly methylated. All the methods are available as ‘differential.coverage’ R package (https://github.com/favorov/differential.coverage).

To identify differentially methylated CpG islands, methylated regions were identified as positional peaks of aligned sequencing reads in the MBD-enriched data using MACS v1.4 software^41,42^. MACS commonly identifies peaks after accounting for both global and local biases using the enriched-to-input fraction. A MACS p-value cut-off (p < 10^−6^) was used to define regions that were methylated. We annotated human genome CpG islands from the UCSC DAS server. Using MACS-processed MBD-Seq data, we calculated the net length of methylated regions that overlap with each CpG island for each sample. For these CpG islands, we calculated the Wilcoxon p-values for association between cases and controls (47 HPV+ OPSCC cases and 25 normal controls) and any MACS signal presence in the CpG island of the sample. All the regions with Wilcoxon p-value > 0.05 were considered significantly methylated.

### EpiTYPER

All tissue samples were collected after informed consent from patients under an approved IRB protocol at the University of Pittsburgh and VA Pittsburgh Health System in accordance to guidelines and regulations of the institution. Quantitative analysis of DNA methylation was carried out using matrix-assisted laser desorption/ionization time-of-flight (MALDI-TOF) mass spectrometry. Briefly, formalin fixed HNSCC samples were obtained from patients undergoing surgery at the University of Pittsburgh Medical Center and VA Pittsburgh Healthcare System under an approved IRB protocol. HPV status was determined by immunohistochemistry for p16 (G175–405, BD Pharmingen, San Diego, CA). DNA was extracted using the Qiagen DNeasy kit according to the manufacturer’s protocol (Qiagen, Germany). Extracted DNA was treated with sodium bisulfite using the EpiTect bisulfite conversion kit according to the manufacturer’s protocol (Qiagen, Germany). Bisulfite treated DNA was then converted into amplicons for EpiTYPER using sequence specific primers (Supplementary table 1). PCR conditions were 5 min at 95 °C, then 1 min at 95 °C, 1 min at 61 °C, 2 min at 72 °C repeated 45 times, then 5 min at 72 °C for TSS and exon 2; reaction conditions were identical for the DRE except 57 °C was used as the annealing temperature. Reactions were carried out in triplicate and run on a 1% agarose gel for confirmation prior to EpiTYPER. Additionally, unmethylated, 50% methylated, and fully methylated control DNA was run with each primer pair as a control for sufficient bisulfite conversion.

Amplicons were prepared using the Sequenom EpiTYPER kit (Sequenom, San Diego, CA). Briefly, amplicons were dephosphorylated with shrimp alkaline phosphatase then amplified using T7 DNA polymerase at 37 °C for 3 hours. Samples were loaded onto a SpectroCHIP bioarray and analyzing using MassARRAY (Sequenom, San Diego, CA). Mass spectra were analyzed using EpiTYPER software and converted to methylation percentage for each CpG. Sequences and CpG’s covered by each amplicon are available in Supplementary Table 2. Triplicates were averaged within each subject. A two-way repeated measures ANOVA with Bonferroni post-hoc tests was used to identify an effect of HPV status on DNA methylation.

### NOK cell line DNA methylation and expression

Normal oral keratinocyte (NOK) cell lines transfected with sequences for the HPV proteins E6, E7, or an empty LXSN vector control^43^ (gift from Dr. Saleem Khan, University of Pittsburgh) were grown in keratinocyte serum free media and three separate passages were used for experiments. RNA was extracted using Trizol (Life Technologies, Carlsbad, CA) and purified using the RNeasy mini kit according to the manufacturer’s protocol (Qiagen, Germany). RNA was reverse transcribed using iScript according to the manufacturer’s protocol (Bio-Rad, Hercules, CA) and used for qRT-PCR with SYBR Green (Bio-Rad, Hercules, CA). Expression of E6 and E7 was confirmed using sequence specific primers: Forward-E6 ATGCACCAAAAGAGAACTGC and Reverse-E6 TTACAGCTGGGTTTCTCTAC; Forward-E7 CAGCTCAGAGGAGGAGGATG and Reverse-E7 GCACAACCGAAGCGTAGAGT. ANO1 expression was studied using primers Forward-ANO1 CTCCTGGACGAGGTGTATGG and Reverse-ANO1 GAACGCCACGTAAAAGATGG and actin was used as a loading control, Forward-Actin AGAGCTACGAGCTGCCTGAC and Reverse-actin AGCACTGTGTTGGCGTACAG. qRT-PCR was run in duplicate using the following reaction conditions: 10 min at 95 °C, then 15 s at 95 °C, 30 s at 60 °C, 30 s at 72 °C repeated 40 times. The difference between ANO1 and actin (ΔCt) was calculated for each sample and normalized to the average of NOK controls (ΔΔCt). Fold change over NOK controls was calculated using the following formula: 2^−ΔΔCt^. DNA methylation analysis of NOK cells was carried out as described in the EpiTYPER methods section.

## Electronic supplementary material

Supplementary data
