# Supplementary material for: DNA methylation regulates TMEM16A/ANO1 expression through multiple CpG islands in head and neck squamous cell carcinoma
Source: Sci Rep. 2017 Nov 9;7:15173. doi: 10.1038/s41598-017-15634-9 (PMC5680248; doi:10.1038/s41598-017-15634-9)
Supplement: Supplementary file 1 — Supplementary data [file 41598_2017_15634_MOESM1_ESM.pdf]

DNA methylation regulates TMEM16A/ANO1 expression through multiple CpG islands in head and neck squamous cell carcinoma

Andrey Finegersh[1,2,], Scott Kulich[3], Theresa Guo[4], Alexander V. Favorov[5,6], Elana J. Fertig[5], Ludmila V. Danilova[5], Daria A. Gaykalova[4], Joseph A. Califano[2,4], Umamaheswar Duvvuri*[1,3]

[1]Department of Otolaryngology, University of Pittsburgh School of Medicine, Pittsburgh, PA, USA

[2]Division of Otolaryngology-Head and Neck Surgery, Department of Surgery, University of California, San Diego, CA, USA

[3]VA Pittsburgh Healthcare System, Pittsburgh, PA, USA

[4]Department of Otolaryngology-Head and Neck Surgery, Johns Hopkins University School of Medicine, Baltimore, MD, USA

[5]Department of Oncology, Johns Hopkins University School of Medicine, Baltimore, MD, USA

[6]Laboratory of Systems Biology and Computational Genetics, Vavilov Institute of General Genetics, RAS, Moscow, Russia

<u>**Supplementary tables and figures**</u>

Supplementary Table 1. EpiTYPER primers

Supplementary Table 2. EpiTYPER amplicon sequences and covered Illumina DNA methylation 450k assay probes

Supplementary Figure 1. Differential expression of ANO1 in methylated and unmethylated OPSCC tumor and normal tissue samples at the DRE and exon 2.

Supplementary Figure 2. Expression of ANO1, cyclin D1, and cortactin in HPV+ OPSCC relative to normal oral mucosa.

Supplementary Figure 3. Minimal expression of ANO1 exon 0 detected in OPSCC and normal oral mucosa using RNA-seq.

Supplementary Figure 4. HPV- samples have hypomethylation of negatively correlated CpGs relative to HPV+ samples on CCND1 and CTTN.

Supplementary Figure 5. Copy number variation enhances the effects of DNA methylation at significantly correlated CpGs.

Supplementary Table 1. EpiTYPER primers

Region	Forward primer	Reverse primer
DRE	aggaagagagTTATTGTTAAGGAAAATGGTAGGTT	cagtaatacgactcactatagggagaaggctACCTAAAAACACTACCCCAAAAAAA
TSS	aggaagagagGAGTGTTGAGTGATAGTAGGAGTTTGTT	cagtaatacgactcactatagggagaaggctAAAACTTTCAATAACCCCACCTAAA
Exon 2	aggaagagagTATTTTTTGATTATGGAAGTAGTTGTT	cagtaatacgactcactatagggagaaggctTCAAACAAAACCACCCCCTAC

Adaptor sequences in lower case

Supplementary Table 2. EpiTYPER amplicon sequences and covered Illumina DNA methylation 450k assay probes

Region	Sequence	Illumina Probe
Distal Enhancer	GATCATTGCTAAGGAAAATGGCAGGTCACCTTTCTTTCAGAC<br>TCAATG**CG**AGGAAGGAATCTTGCCTCT**CG**TCTCCTTTTACAG<br>TACAG**CG**CTGAGGTGGGGG**CG**GGGAAGGAGACC**CG**GCAGGAA<br>ACATGTCTTTGTGGGTTTGC**CG**CT	cg20023120
TSS	GCAGAGATCCAGACTCCAATCTCAGAAGCTTCACTCCCCC**CG**<br>GAGG**CG**GAGGTGA**CG** **CG**CCACAGAAG**CG**CCTGCCACT**CG**GGG<br>ACTTTATTTCAGGTGGGGCTATTGAAAGTTCCCCCCTTTCAC<br>ACC**CG**GTATGAAAA	cg01120391<br>cg18141744
Exon 2	GACCACCCCCTGC**CG**GGCAAGGGGG**CG**T**CG**CTGGATGCAGGC<br>T**CG**GGGGAGCCCC**CG**ATGGACTACCA**CG**AGGATGACAAG**CG**C<br>TTCCGCAGGGAGGAGTA**CG**AGGGCAACCTCCTGGAGG**CG**GGC<br>CTGGAGCTGGAG**CG**GGA**CG**AGGA**CG**TAACTATCTCACTG**CGC**<br>**G**CTGTTTGTGGGGGGTGGGGGGTGGGCTG**CG**TGGGGGGCAGT	cg05846633<br>cg13455439

Supplementary Figure 1. Differential expression of ANO1 in methylated and unmethylated OPSCC tumor and normal tissue samples at the DRE and exon 2.

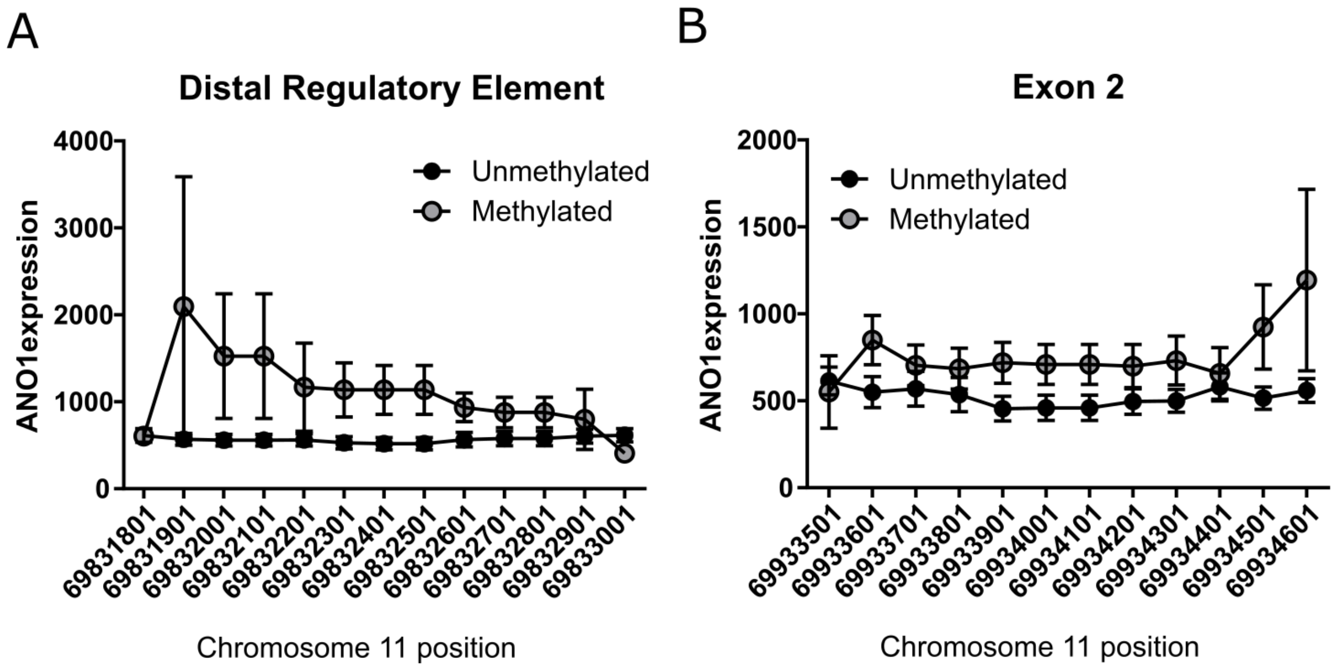

Effect of DNA methylation at individual CpGs on expression of ANO1 for 72 samples in the OPSCC dataset (47 OPSCC tumors and 25 normal tissue samples) at (A) the distal regulatory element and (B) Exon 2. Mean +/- SEM presented relative to chromosome position.

Supplementary Figure 2. Expression of ANO1, cyclin D1, and cortactin in HPV+ OPSCC relative to normal oral mucosa.

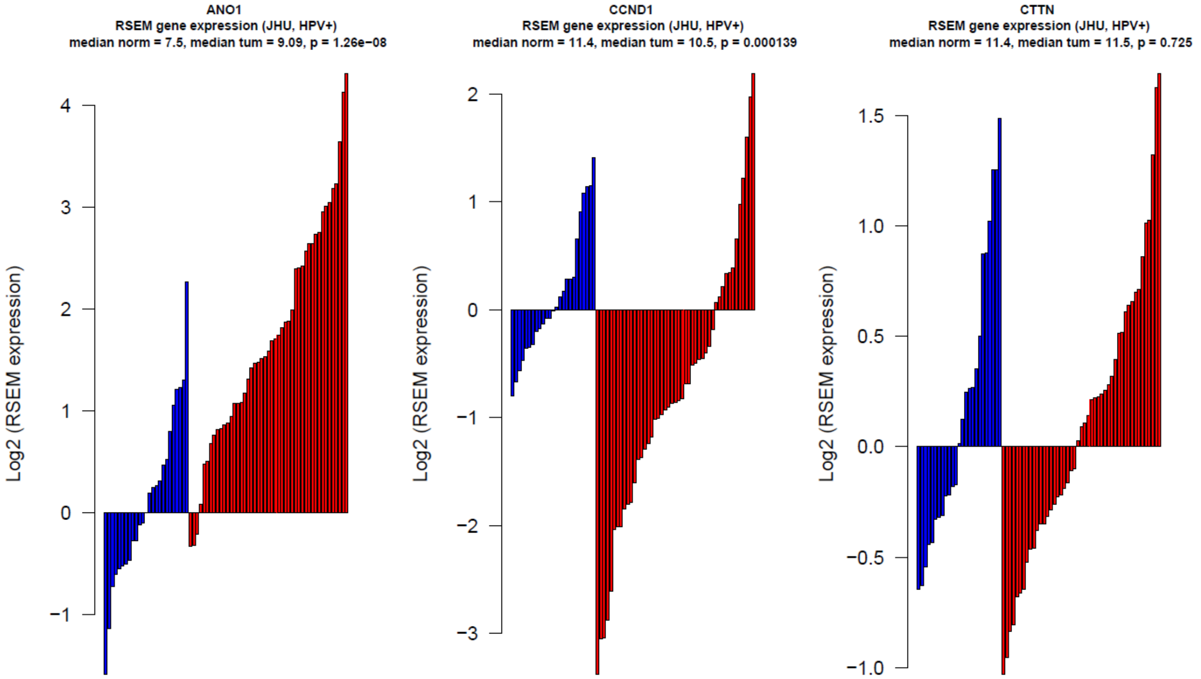

Expression plot of ANO1, cyclin D1 (CCND1), and cortactin (CTTN) in HPV+ OPSCC (n = 47) compared to normal oral mucosa from control subjects (n = 25). There was significantly increase expression of ANO1, significantly decreased expression of CCND1, and no change in expression of CTTN in OPSCC relative to normal mucosa. Blue bars are normal tissue samples and red bars are OPSCC tumor samples.

Supplementary Figure 3. Minimal expression of ANO1 exon 0 detected in OPSCC and normal oral mucosa using RNA-seq.

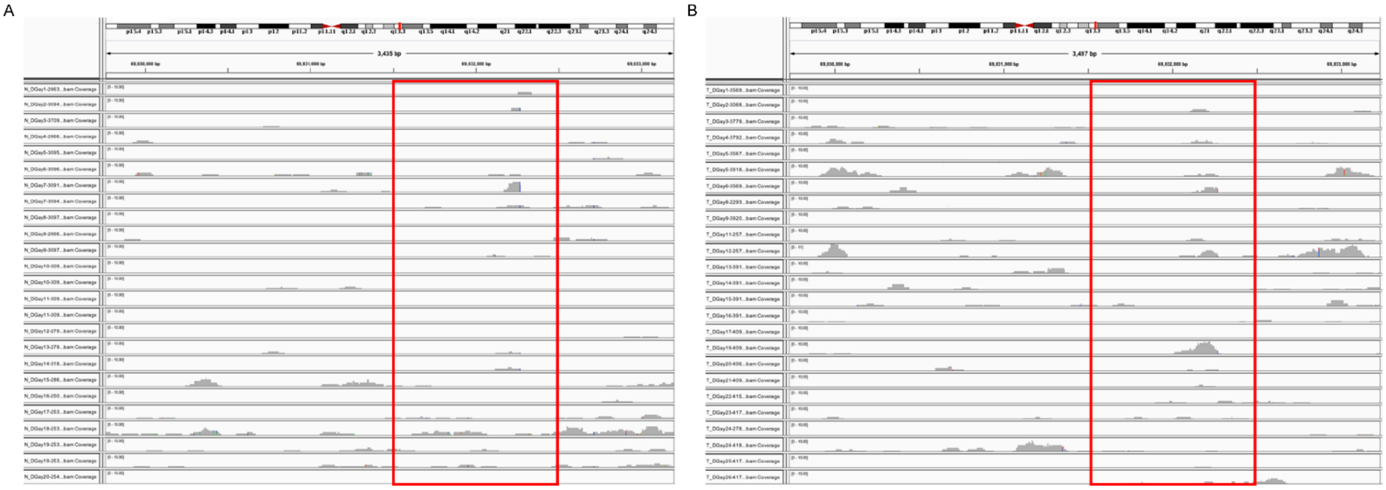

Representative RNA-seq IGV read counts for the ANO1 5’ distal regulatory element and exon 0 (chr11:69831572-69832484) in (A) normal oral mucosa and (B) OPSCC tumors. Red boxes indicate where IGV read counts were studied.

Supplementary Figure 4. HPV- samples have hypomethylation of negatively correlated CpGs relative to HPV+ samples on CCND1 and CTTN.

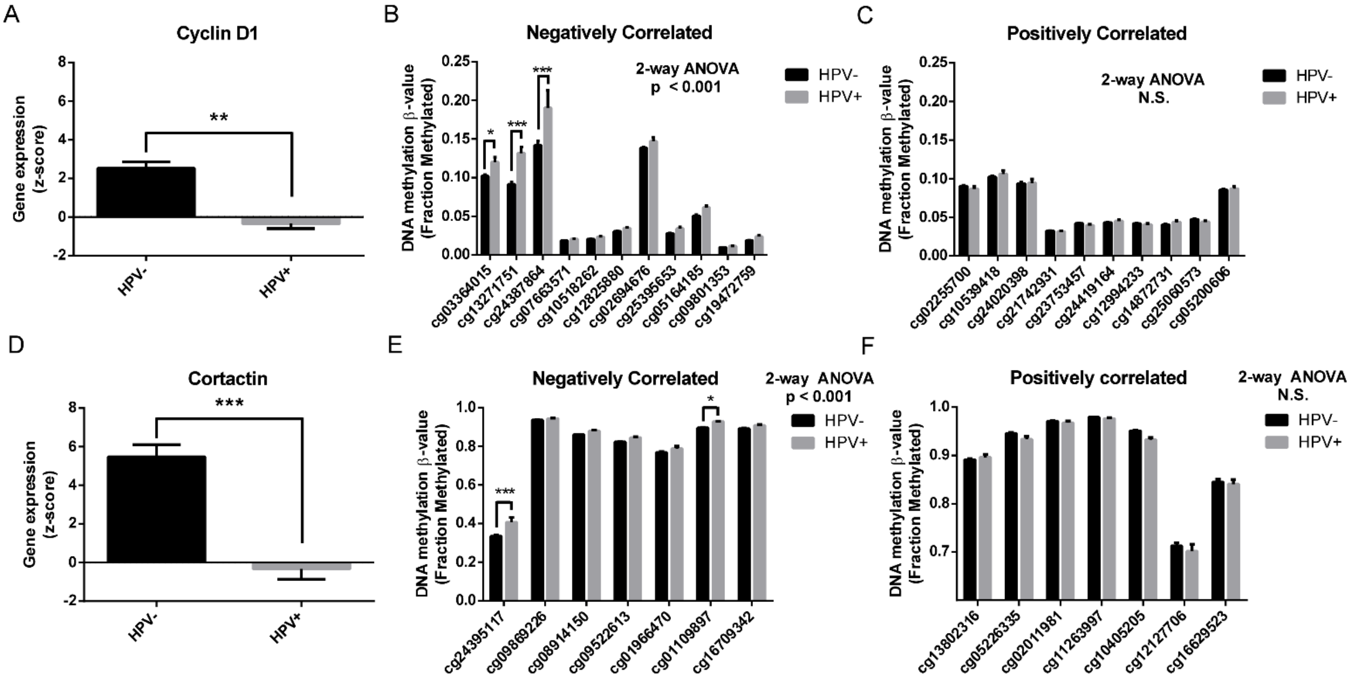

DNA methylation in HPV+ (n = 34) and HPV- (n = 241) samples was compared at the Cyclin D1 (CCND1) and Cortactin (CTTN) promoters. (A,D) There was significantly increased expression CCND1 and CTTN in HPV- relative to HPV+ HNSCC samples. Using a two-way repeated measures ANOVA we found that (B,E) at negatively correlated CpGs there was a significant effect of HPV infection on DNA methylation and Bonferroni post-hoc tests revealed significantly decreased methylation in HPV- samples. (C,F) At positively correlated CpGs there was no effect of HPV infection on DNA methylation. Data presented as mean ± SEM. *p < 0.05, **p < 0.01, ***p < 0.001.

Supplementary Figure 5. Copy number variation enhances the effects of DNA methylation at significantly correlated CpGs.

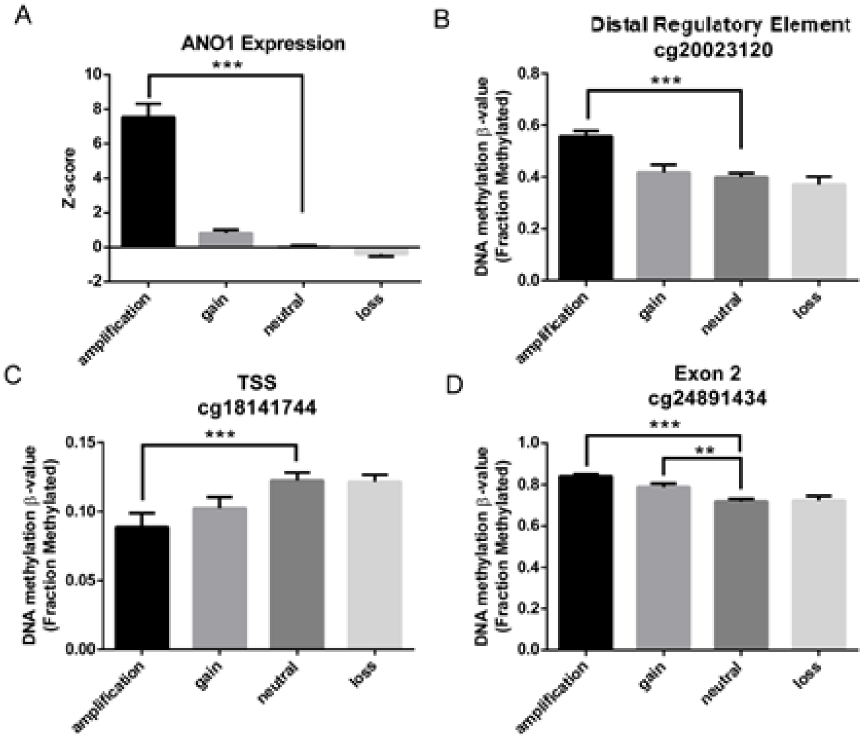

HNSCC samples were classified by the TCGA as having ANO1 copy number amplification (n = 79), gain (n = 48), neutrality (n = 119), or loss (n = 29) and ANO1 expression and DNA methylation were analyzed at the most correlated Illumina probe across three CpG islands. Relative to neutral samples, (A) amplified samples had significantly increased expression, (B) amplified samples had significantly increased DNA methylation of the positive correlated cg200232120, (C) amplified samples had significantly decreased DNA methylation at the negatively correlated CpG cg18141744, and (D) amplified and gain samples had significantly increased DNA methylation of the positively correlated CpG cg24891434. Data presented as mean ± SEM. $^{**}p < 0.01$, $^{***}p < 0.001$
